# Genome-wide identification of conditionally essential genes for growth in the presence of sulfamethoxazole and trimethoprim in sulfamethoxazole- and trimethoprim-resistant *Escherichia coli*

**DOI:** 10.1128/spectrum.00603-26

**Published:** 2026-07-29

**Authors:** Sajid Nisar, Mosaed S. Alobaidallah, Sandra M. Wellner, Nader Abdelmalek, Mauro M. Saraiva, Line E. Thomsen, John E. Olsen

**Affiliations:** 1 Department of Veterinary and Animal Sciences, Faculty of Health and Medical Sciences, University of Copenhagen 555025, Frederiksberg, Denmark; 2 Department of Clinical Laboratory Sciences, College of Applied Medical Sciences, King Saud bin Abdulaziz University for Health Sciences 841812, Jeddah, Saudi Arabia; 3 King Abdullah International Medical Research Center, Jeddah, Saudi Arabia; 4 Ministry of the National Guard—Health Affairs, Jeddah, Saudi Arabia; 5 Unit of Microbiology and Virology, Department of Biomedical Sciences, University Hospital of Sassari, University of Sassari 9312, Sassari, Italy; 6 Department of Pathology, Reproduction, and One Health, Universidade Estadual Paulista-Unesp-FCAV, Jaboticabal, Brazil; University of Minnesota Twin Cities, Minneapolis, Minnesota, USA

**Keywords:** antibiotic resistance, TraDIS, conditionally essential genes, sulfonamides, TMP

## Abstract

Resistance to sulfonamides (SULs) and trimethoprim (TMP) in *Escherichia coli* threatens their clinical relevance. Beyond known resistance mechanisms, little is understood about the cellular responses that enable resistant *E. coli* to grow under these antibiotic stresses. This study aimed to identify genes that support bacterial growth under SUL and TMP stress. Two saturated transposon mutant libraries were constructed in resistant *E. coli* MG1655 harboring either *dfrA1* or *sul2*. They were grown with and without 1/2 and 1/4 minimum inhibitory concentration (MIC) of sulfamethoxazole (SMX) or TMP, and mutant depletion was assessed via transposon-directed insertion-site sequencing. At 1/2 MIC, 36 and 89 genes were identified as conditionally essential during SMX and TMP exposure, while 5 and 2 genes were classified as conditionally essential at 1/4 MIC. Genes identified as conditionally essential at 1/4 MIC were also important at 1/2 MIC. Conditionally essential genes belonged to lipopolysaccharide biosynthesis, peptidoglycan metabolism, energy production, membrane integrity, phosphate metabolism, and stress responses, highlighting the role of these factors in maintaining cell stability under SMX and TMP stress. Validation with 10 conditionally essential genes (*apaH, mtn, surA, waaO, nlpI, prc, wzxE, fadR, degP,* and *tpiA*) showed that deletion mutants indeed exhibited growth defects and two- to eightfold reductions in MIC under antibiotic stresses compared to their parent strains. This study highlights cellular responses to SMX and TMP under antibiotic stress, and it has identified a list of genes whose products may serve as potential helper drug targets to resensitize resistant *E. coli* to SMX and TMP treatments.

**IMPORTANCE:**

Sulfonamides (SULs) and trimethoprim (TMP) are broad-spectrum antimicrobials. They are commonly used to treat infections in both humans and animals. Resistance against SUL and TMP is widespread in pathogenic bacteria, and there is a need to overcome this problem. One possibility is to target the cellular mechanism by which the resistant bacteria adapt to growth in the presence of the antimicrobials. In this study, we identify the genes, besides the resistance genes, which enable resistant *Escherichia coli* to grow in the presence of SUL and TMP. We further show that knocking out many of these genes attenuates the resistant *E. coli* for growth during SUL and/or TMP stress, irrespective of which SUL- or TMP-resistant gene the bacteria carry. The gene products of these genes may serve as potential helper drug targets to resensitize resistant *E. coli* to sulfamethoxazole and TMP treatments.

## INTRODUCTION

The escalating crisis of antimicrobial resistance (AMR) threatens both human and animal health worldwide, causing an estimated 4.71 million deaths related to bacterial resistance in 2021, including 1.14 million deaths directly attributable to AMR. The study noted a significant increase in deaths associated with resistant Gram-negative bacteria, including *Escherichia coli*, between 1990 and 2021 (1). If no actions are taken, by 2050, AMR-related deaths could exceed 10 million annually, with the heaviest burden likely in South Asia and Latin America (2).

Sulfonamides (SULs) and trimethoprim (TMP) are synthetic antibiotics belonging to the sulfa and diaminopyrimidine drug classes, respectively. They target bacterial metabolism by sequentially inhibiting the *de novo* synthesis of folate, ultimately impairing DNA/RNA formation, which leads to cell cycle arrest (3). SUL drugs act as competitive inhibitors of *folP*-encoded dihydropteroate synthase (DHPS) by mimicking para-aminobenzoic acid (PABA), a major precursor in folate formation in bacteria (4). When SULs bind to DHPS, they block the condensation of PABA and pteridine to dihydropteroic acid, which hinders the formation of downstream folate intermediates, even when SULs are used as monotherapy (4, 5). In the absence of SUL, dihydropteroic acid is converted to dihydrofolate (DHF) through the action of the *folC*-encoded dihydrofolate synthase. TMP prevents the formation of folate by blocking the *folA*-encoded dihydrofolate reductase (DHFR), which participates in the reduction of dihydrofolate to tetrahydrofolate (THF), leading to a scarcity of purines and thymidine and also ultimately affecting DNA/RNA synthesis (4). The broad-spectrum bacteriostatic effect of these antibiotics has contributed to their extensive use in veterinary and human medicine (6, 7).

Similar to other classes of antimicrobials, increased resistance has compromised clinical use of SUL and TMP. SUL resistance in various Gram-negative enteric pathogenic bacteria, including *E. coli*, is mainly mediated by horizontal transfer of plasmid-encoded resistance genes, typically *sul1, sul2, sul3,* and *sul4* (8–10), and to a lesser extent by a mutation in the chromosomally encoded *folP* gene, leading to different variants of DHPS enzymes with no or negligible affinity for sulfa drugs (11). Resistance to TMP is mediated by genes of the *dfrA* and *dfrB* families, which encode diverse variants of DHFRs having less or no affinity for TMP. The *dfrA* gene family encodes large proteins (152–189 amino acids), and at least 52 diverse types of *dfrA* genes have been described (12, 13). In comparison, the *dfrB* gene family encodes shorter proteins (70–78 amino acids), and until now, 21 *dfrB* genes have been documented (14).

The increasing resistance in bacteria makes it highly interesting to seek ways to repurpose the use of traditional antibiotics in clinical practice, including sulfamethoxasole (SMX) and TMP (15). To achieve this, a comprehensive understanding of the molecular basis of SMX and TMP resistance is needed. Surprisingly, little is known about the system-level changes that occur in SMX- and TMP-resistant *E. coli* when they are exposed to therapeutic doses of these antibiotics.

Transposon-directed insertion site sequencing (TraDIS) (16) and related transposon insertion sequencing methods have revolutionized AMR research by allowing genome-wide detection of conditionally essential genes under selective pressure. TraDIS studies in *E. coli*, *Klebsiella pneumoniae*, *Staphylococcus aureus*, and *Streptococcus pyogenes* have identified genes associated with fitness and resistance in response to aminoglycosides, β-lactams, chloramphenicol, macrolides, colistin, and ciprofloxacin (17–23). In this study, we employed TraDIS to systematically identify genes that were conditionally essential for growth in the presence of SMX and TMP in resistant *E. coli*. We hypothesized that, apart from the known resistance genes, other genes of importance to structural and metabolic functions would be needed for growth in the presence of SMX (512 and 1,024 mg/L) and TMP (750 and 1,500 mg/L), corresponding to 1/4 and 1/2 of the minimum inhibitory concentration (MIC), respectively, despite such concentrations being sub-inhibitory to the resistant bacteria.

To evaluate this hypothesis, we created two highly saturated transposon mutant libraries in *E. coli* MG1655 resistant to SMX and TMP. These libraries were grown under selective pressure at 1/2 MIC and 1/4 MIC of these drugs. Genome-wide screening confirmed known resistance factors and identified new genetic dependencies that enable bacteria to grow under SMX and TMP stress. Selected conditionally essential genes were validated with deletion mutants, many of which showed significantly reduced fitness. This approach identified a list of vulnerabilities that could be targeted by helper compounds to re-sensitize the bacteria to SMX and TMP.

## MATERIALS AND METHODS

### Bacterial strains and culture conditions

Bacterial strains and plasmids are listed in Table S1. The base strain used in this study was *E. coli* K-12 MG1655 (24) due to its well-characterized genome and extensive use in previous TraDIS studies investigating antibiotic stress responses (17, 18, 20, 21). Strains were grown overnight on Luria-Bertani (LB) agar plates (Sigma-Aldrich, Copenhagen, Denmark) or in LB broth with continuous shaking at 180 rpm at 37°C. When relevant, the growth media were supplemented with appropriate antibiotics (Sigma-Aldrich, Copenhagen, Denmark), including TMP (20 mg/L), SMX (64–256 mg/L), chloramphenicol (CHL) (25–50 mg/L), and kanamycin (KAN) (50 mg/L). Strains harboring the temperature-sensitive plasmid pKD46 were cultured at 29°C in LB medium supplemented with gentamicin (20 mg/L).

### Preparation of electrocompetent *E. coli* cells and transformation

Electrocompetent cells of *E. coli* MG1655 were prepared as previously described (25) with minor modifications. Briefly, 1.5 mL of *E. coli* MG1655 overnight culture was inoculated into 150 mL of LB broth and grown to an OD_600_ of 0.4–0.6. Next, the cells were collected by centrifugation at 7,200 × *g* for 15 min, washed twice with sterilized ice-cold Milli-Q water, and three times with sterilized 10% ice-cold glycerol. Finally, the cells were resuspended in 200 μL of 10% ice-cold glycerol. Aliquots of 70 µL were stored at −80°C for future use.

For transformation, 70 µL of electrocompetent cells and 1–5 µL of PCR product or plasmid were mixed and incubated on ice for 10 min. The mixture was then transferred to an ice-cold 2 mm gap electroporation cuvette and electroporated immediately using an Eporator electroporator (Eppendorf, Hamburg, Germany) with the p2 program set at 12.5 kV/cm, 600 Ω, and 25 µF. Next, the cells were immediately recovered in 950 μL of prewarmed Super Optimal broth with Catabolite repression (SOC) medium (Thermo Fisher Scientific, Denmark) and incubated at 37°C (or 29°C for pKD46-containing cells) with vigorous shaking at 180 rpm for 1.5 h. Following transformation, the transformed cells (50–100 µL) were plated on LB agar plates containing the appropriate antibiotic. Plates were incubated overnight at 37°C, except for strains carrying a temperature-sensitive plasmid (e.g., pKD46), which were incubated at 29°C. The next day, four to five colonies were randomly selected to confirm the presence of the resistance plasmid or gene by colony PCR with primers listed in Table S2 and re-streaked on selection plates. Glycerol stocks were prepared for the confirmed transformants, with 25% glycerol stored at −80°C.

### Construction of plasmids harboring sulfonamides and trimethoprim resistance genes

Plasmids harboring TMP and SMX resistance genes were constructed essentially as previously described (21). Briefly, PCR amplification with overhang primers was used to amplify the TMP resistance genes (*dfrA1*, *dfrA5*, and *dfrA12*) and sulfa resistance genes (*sul1*, *sul2*, and *sul3*) from the clinical *E. coli* isolates 110331-1, AV41, 113038-4, and 113864-1, selected from a previous study (26). Details on strains and genes can be found in Table S1. Similarly, the backbone (2,520 bp) of the pACYC184 plasmid, including the origin of replication with chloramphenicol resistance gene but excluding the tetracycline resistance gene, was amplified using overhang primers. All the primers were designed using the NEB online primer design tool (New England BioLabs, USA). PCR amplifications were carried out using Q5 master mix (New England BioLabs, USA).

The amplified sulfa and TMP resistance genes were assembled into the pACYC184 backbone using the NEBuilder HiFi DNA Assembly Master Mix following the manufacturer’s protocol (New England BioLabs, USA). Six plasmids were constructed (Fig. S1 and S2) and then transformed into chemically competent *E. coli* DH5α cells following the manufacturer’s instructions (New England BioLabs, USA). Transformants were selected on selection plates with the appropriate antibiotics (SMX, TMP, and CHL) and further validated by colony PCR. Plasmids from positive clones were purified using the QIAprep Spin Miniprep kit (QIAGEN Nordic, Denmark) and sequenced by Sanger sequencing (Macrogen Europe) to confirm the presence and integrity of the cloned resistance genes. Following sequencing, the purified plasmids were transformed into electrocompetent MG1655 cells, as described above. *E. coli* MG1655 strains harboring *sul1*, *sul2*, or *sul3* plasmids will be referred to as sulfa-resistant parent strains, while those carrying *dfrA1*, *dfrA5*, or *dfrA12* will be referred to as TMP-resistant parent strains. Primers used for plasmid construction, sequencing, and colony PCR are listed in Table S2. Plasmid sequences are available at http://doi.org/10.17894/ucph.7457f751-c7d5-4823-a372-9d365f5b46ca.

### Assessment of minimum inhibitory concentration

MICs of SMX and TMP were determined using the broth microdilution method following CLSI guidelines (27). SMX was tested in the range of 1–4,096 mg/L and TMP from 0.25 to 6,000 mg/L. Results were interpreted according to recommendations given by both CLSI and EUCAST, stating that pinpoint growth at the bottom of the wells should be disregarded, and that the MIC is defined as the lowest concentration that inhibited ≥80% of growth compared to the growth control (27, 28). The MIC was determined using three biological replicates, each with four technical replicates.

### Growth curve analysis

Bacterial suspensions for growth analysis were prepared in the same way as the MIC testing. Briefly, a 0.5 McFarland bacterial suspension (approximately 1.5 × 10^8^ colony-forming units [CFUs]/mL) was prepared using a Sensititre nephelometer (Thermo Fisher Scientific, Denmark) and diluted to ~10^6^ CFU/mL by adding 0.1 mL of the bacterial suspension to 9.9 mL LB broth. Subsequently, 125 µL of the bacterial suspension (~10^6^ CFU/mL) was mixed with 125 µL of LB medium containing either specified concentrations of antibiotics (e.g., 1,500 mg/L TMP and 1,024 mg/L SMX) or no antibiotics in a 100-well honeycomb plate (Oy Growth Curves Ab Ltd., Finland). The first and last columns and rows served as negative controls. OD_600_ values were monitored every 20 min using Bioscreen C (Oy Growth Curves Ab Ltd., Finland) under medium and continuous shaking for 24 h at 37°C. Experiments were conducted with three biological and three technical replicates. Growth curves with standard error and mean were generated using GraphPad Prism 10.3.0 (GraphPad Software, USA).

### Construction and validation of TraDIS input libraries

Electrocompetent cells of the sulfa-resistant parent strain [MG1655/pACYC184_ΔTet^R^ (CHL^R^)_*Sul2* (SULFA^R^)] and the TMP-resistant parent strain [MG1655/pACYC184_ΔTet^R^ (CHL^R^)_*dfrA1* (TMP^R^)] were made as described above. Then, 1 µL of EZ-Tn5 < KAN-2 > Tnp Transposome (Epicentre, Nordic Biolabs, Sweden) was mixed with a 70 µL aliquot of electrocompetent cells, and the mixture was electroporated in a 1 mm cuvette using Eporator electroporator (Eppendorf, Hamburg, Germany) at P1 program set at 1.8 kV. Afterward, the cells were instantly recovered in 950 μL of pre-warmed SOC medium and incubated at 37°C with vigorous shaking at 180 rpm for 1.5 h. Following incubation, aliquots of 100 µL of the cell suspension were spread onto 10 large LB agar plates (15 cm) containing 50 mg/L KAN (Sigma-Aldrich, Denmark). After overnight incubation at 37°C, KAN-resistant colonies from each plate were harvested and resuspended in 1–2 mL of LB broth, supplemented with 20% glycerol. This process was repeated until at least 200,000 mutants were achieved for each library. Colonies from each batch of mutants from all plates were pooled into the same 50 mL tube for the respective library and stored at −80°C. This process resulted in the construction of two high-density transposon mutant libraries in the sulfa- and TMP-resistant *E. coli* strains. CFU count of each input library was determined to be 1–2 × 10^11^ CFU/mL by the plate spread dilution method, and aliquots containing approximately 5 × 10^9^ CFU in 1 mL LB supplemented with 20% glycerol were prepared and stored at −80°C.

Random amplification of transposon ends PCR was performed on 12 randomly selected KAN-resistant colonies from each library to validate the random insertion of the Tn5-KAN-2 transposon following the previously described protocol (29).

### Construction of TraDIS output libraries

**Fig 1:**
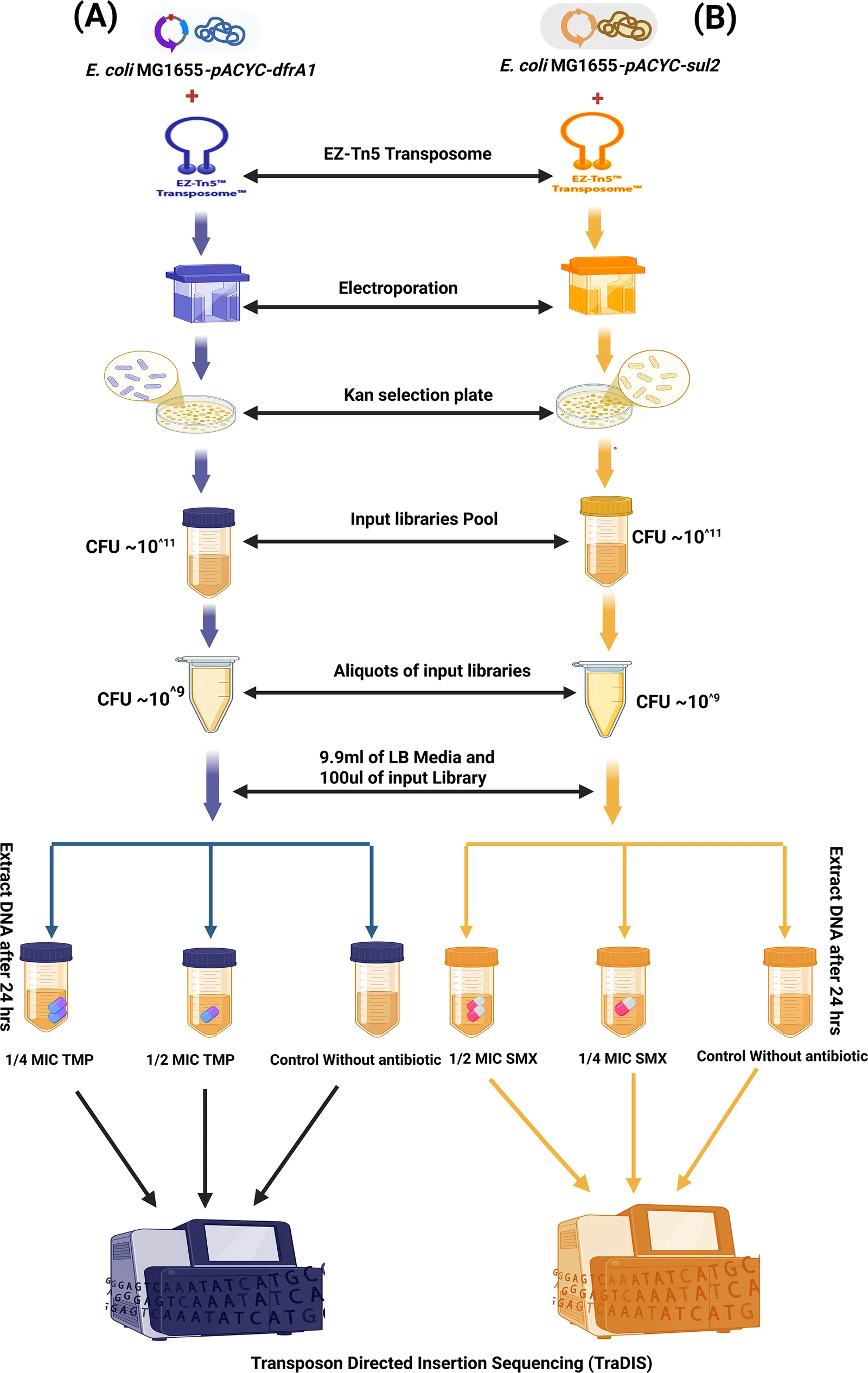
Overview of the TraDIS experimental workflow used in the study. (**A**) A transposon mutant library was constructed in *E. coli* MG1655 harboring the *dfrA1* gene, conferring TMP resistance. (**B**) A parallel library was generated in MG1655 carrying *sul2*, conferring SUL resistance. Input libraries were exposed to TMP or SMX at 1/2 MIC, 1/4 MIC, and no drug (control) and incubated for 24 h. After incubation, cells were harvested for DNA extraction, and Illumina sequencing was performed. Transposon insertion sites were then identified and analyzed using the Bio-TraDIS pipeline.

One hundred microliters of each input Tn-library, containing approximately 5 × 10^9^ mutants, was inoculated into 9.9 mL of LB medium (~1 × 10^7^ final CFU) supplemented with SMX at 1,024 or 512 mg/L, TMP at 1,500 or 750 mg/L, or LB medium without these antibiotics as a control. The concentrations corresponded to 1/2 MIC and 1/4 MIC of each drug. The libraries were grown at 37°C at 180 rpm for 24 h. After incubation, CFUs were quantified to assess cell density. The experiments were carried out in duplicate, generating six output libraries for each input library, resulting in a total of 12 output Tn libraries. An overview of the total workflow is shown in Fig. 1.

### Preparation of TraDIS input and output libraries for sequencing

The libraries were prepared for sequencing following a previously established protocol (25) with small modifications. In brief*,* 500 μL aliquots of each output and input library (containing ~3 × 10^9^ mutants) were spun down at 13,000 × *g* for 2 min. The cell pellets were washed twice with Dulbecco’s phosphate-buffered saline, and bacterial genomic DNA was isolated using the GenElute Bacterial Genomic DNA Kit (Sigma-Aldrich, Denmark) according to the manufacturer’s guidelines, with a final elution in 100 µL of UltraPure water (Thermo Fisher Scientific, Denmark). NanoDrop and Qubit dsDNA HS Assay Kit (Thermo Fisher Scientific, Denmark) were used to assess DNA quality and quantity, and only high-quality samples were processed for sequencing (Table S3). For TraDIS sequencing, 3–5 µg of DNA per library was fragmented to ~300 bp using a Covaris M220 (Covaris, Woburn, MA, USA).

Library preparation steps for sequencing were performed according to the published protocol (30), and the prepared DNA libraries were then sequenced using a MiSeq reagent kit v.2 (50 cycles) by the Illumina MiSeq platform as previously described (25). A total of 16 samples were sequenced, including 12 output libraries and 4 input libraries. The primers used for sequencing are listed in Table S4.

### TraDIS data analysis

Bio-Tradis pipeline (https://github.com/sanger-pathogens/Bio-Tradis) (30) was used to analyze TraDIS sequencing data with minor adjustments as previously described (25). A custom *fq2bam.pl* script was used to process raw reads by relocating the transposon tag to the front of the reads and changing these files into SAM format. SAM tools were used to convert the SAM files into BAM format, and subsequently, the resulting files were processed with check_tradis_tags and add_tradis_tags to annotate Tn insertions. The resultant BAM files were converted back to FASTQ format for further analysis. Afterward, the bacteria-tradis mapping pipeline was used to filter reads containing transposon tags and remove these tags. Subsequently, with the help of the SMALT tool (https://www.sanger.ac.uk/tool/smalt-0/), the trimmed reads were mapped to the reference genome MG1655 U00096.3 and relevant plasmid sequences to determine unique transposon insertion sites (UISs) per gene and read counts. Visualization of UISs and creation of a DNA circular plot were performed using Artemis and Artemis DNA Plotter (version 18.2.0). Next, *tradis_essentiality.R* script was used to evaluate gene essentiality across the genome, where the insertion site index, IID (transposon insertions per gene normalized by gene length), was calculated to measure the insertion frequencies between genes of different lengths. Subsequently, genes were categorized as “essential,” “non-essential,” or “ambiguous” by the gamma distribution model. Differential gene fitness analysis was conducted among control and treated conditions using *tradis_comparison.R*, where read counts, log_2_ fold change (log_2_[FC]), and *q*-values were calculated to identify conditionally essential genes. Genes were considered conditionally essential if log_₂_FC ≤ −2 and *q* ≤ 0.01 under antibiotic treatment. Output lists from *tradis-comparison.R*, including read counts, log_2_(FC), *P*-values, and *q*-values, are presented in Table S5.

### Functional categorization of conditionally essential genes

Conditionally essential genes identified under SMX and TMP treatment were functionally categorized based on their established biological roles, using information from EcoCyc (https://ecocyc.org) (31) and UniProt (https://www.uniprot.org) (32). Enrichment analyses were performed using STRING analysis (https://string-db.org) (33).

### Targeted deletion of conditionally essential genes for TraDIS data validation

Lambda Red mediated recombination was used to construct mutants in the wild-type *E. coli* MG1655 strain by knocking out selected conditionally essential genes (*degP, nlpI, prc, fadR, wzxE, surA, apaH, mtn, tpiA,* and *waaO*) using pKD46 and pKD4 plasmids, following previously described protocols (34).

The correct insertion of the KAN-resistance cassette was confirmed by colony PCR using a three-primer set PCR strategy. The first set used both primers outside the cassette, the second set used one primer inside the KAN-resistance cassette and one upstream (same forward primer as set one), and the third set used one primer inside the KAN-resistance cassette and one downstream (same reverse primer as set one). Subsequently, SUL- and TMP-resistant plasmids were transformed into the knockout mutants as mentioned above. Primers used for knockout construction and verification of the cassette are listed in Table S6.

### Competitive growth assay to assess the fitness of knockout mutants

Fifty microliters of 0.5 McFarland standard of each strain (parent and knockout mutant strains) was added to 10 mL of LB medium containing either antibiotics with final TMP 1,500 mg/L and SMX 1,024 mg/L or no antibiotics (control group). The tubes were incubated at 37°C at 180 rpm for 24 h. After 24 h of incubation, the CFU was determined to assess the relative abundance of the knockout mutant strain after growth with and without antibiotics. The parent-resistant strains conferred CHL resistance, and the knockout mutant strain carried both CHL and KAN resistance. Therefore, to determine the relative abundance of the knockout mutant strain by CFU, LB agar plates supplemented with CHL (25 mg/L) were used to enumerate the total bacterial count (parent strain + knockout mutant strain), and plates containing KAN (50 µg/mL) were used to count only the knockout mutants. The relative abundance of the knockout mutants was calculated using the formula: % Relative abundance of knockout mutant = (CFU on KAN plate/CFU on CHL plate) × 100.

Statistical analysis was performed using two-way ANOVA (mixed model) with row-wise multiple comparisons in GraphPad Prism 10.3.0. For each mutant, significant differences in relative abundance between treated and control conditions were assessed to determine conditional essentiality, with significance levels defined as *P < 0.05, **P < 0.01, ***P < 0.001, and ****P < 0.0001.

### Homology assessment of conditionally essential proteins

The amino acid sequences of the gene products of *apaH, nlpI, degP, tpiA, surA, wzxE, fadR, mtn, prc*, and *waaO* were retrieved from Artemis. The sequences were compared to the proteomes of humans (Homo sapiens, taxid: 9606) and other animals, considering that SUL and TMP are used in both human and veterinary medicine. The target animal species included cat (*Felis catus*, taxid: 9685), dog (*Canis lupus* familiaris, taxid: 9615), pig (*Sus scrofa*, taxid: 9823), cow (*Bos taurus*, taxid: 9913), horse (*Equus caballus*, taxid: 9796), sheep (*Ovis aries*, taxid: 9940), goat (*Capra hircus*, taxid: 9925), chicken (*Gallus gallus*, taxid: 9031), and water buffalo (*Bubalus bubalis*, taxid: 89462).

Homology analyses were performed using NCBI’s Protein BLAST tool (https://blast.ncbi.nlm.nih.gov/Blast.cgi; accessed 28 March 2025) following a method similar to that described by Alobaidallah et al. (18). Proteins that showed no significant similarity to human or animal proteins using an *E*-value cutoff of 10 × 10⁻^10^ were classified as non-homologous (35). In addition to sequences from humans and animals, the sequences were also compared by BLAST against bacterial protein databases to assess conservation among other bacterial species.

## RESULTS

### MIC determination for *E. coli* MG1655 carrying different *sul* and *dfrA*-resistance genes

The SUL-resistant strain carrying *sul1* exhibited SMX MIC of 1,024 mg/L, while strains carrying *sul2* and *sul3* showed SMX MICs of 2,048 mg/L. TMP-resistant strains carrying *dfrA1*, *dfrA5*, and *dfrA12* genes all exhibited a TMP MIC of 3,000 mg/L. These values are consistent with previous reports of high-level resistance to SUL (MICs of 2,048–4,096 mg/L) and trimethoprim (MICs > 2,048 mg/L) in *sul*- and *dfrA*-positive isolates (36–39). The clinical ETEC isolates, from which these resistance genes were originally amplified, displayed identical resistance levels.

### Genome-wide coverage and bioinformatics assessment of TraDIS libraries

Bioinformatics analysis of the sulfa- and TMP-resistant transposon-mutant libraries confirmed comprehensive genome coverage, containing 501,096 and 462,355 distinct Tn insertions, respectively, with an average insertion density of one insertion per 11 base pairs (Table S7). TraDIS-bioinformatics analysis revealed high-quality sequence mapping, with 81.08%–90.20% of sequencing reads precisely aligning to the *E. coli* MG1655 reference genome (U00096.3) (Table S7). Insertions were found to be well distributed over the whole chromosome (Fig. S3). An analysis of the SMX- and TMP-resistant input libraries revealed 393 and 389 genes classified by TraDIS as essential in the two libraries, of which a set of 335 genes were essential for growth in both libraries (Tables S8 to S10).

### TraDIS-based identification and functional classification of conditionally essential genes

**Fig 2:**
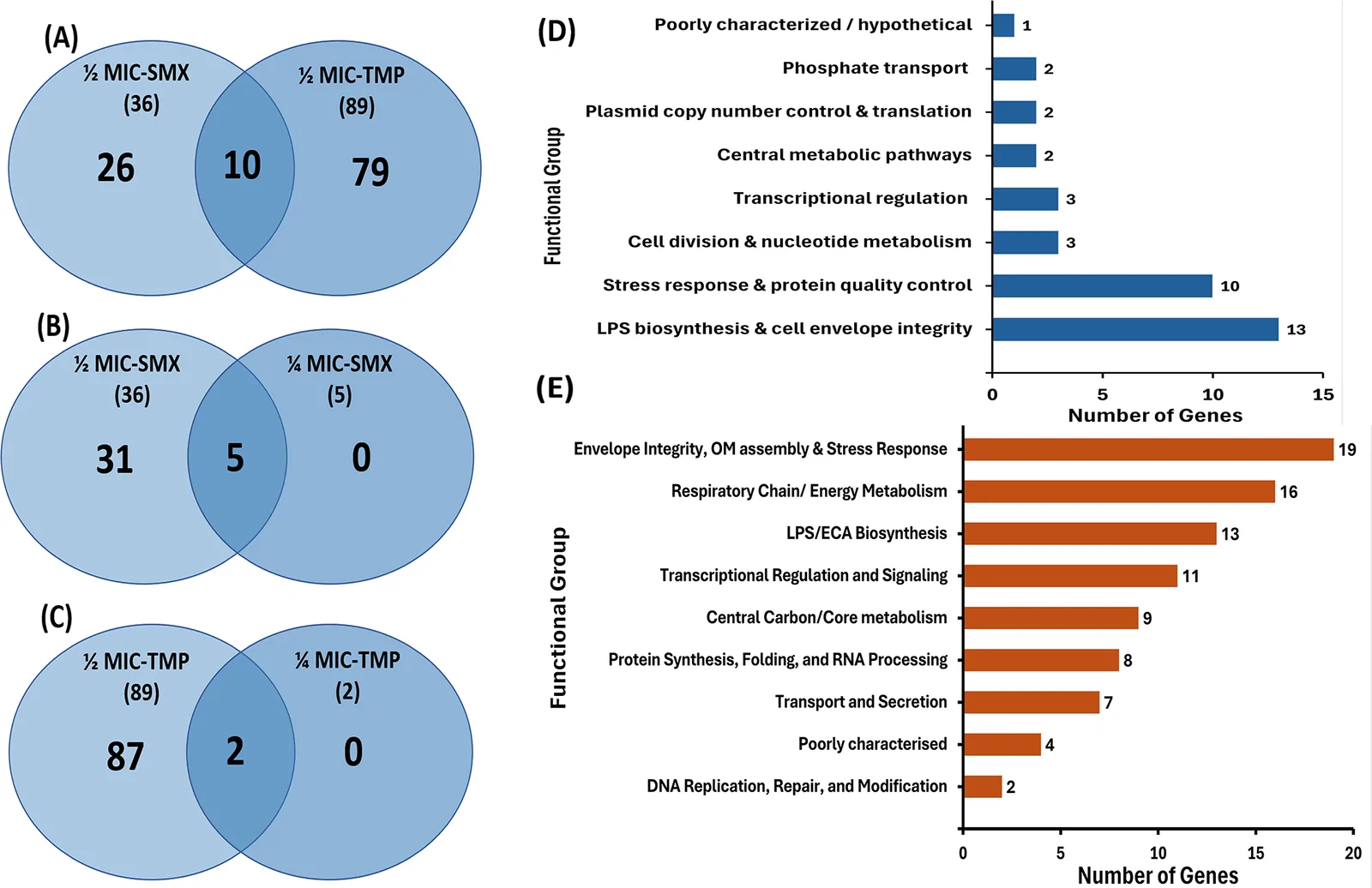
Conditionally essential genes identified in sulfonamide- and trimethoprim-resistant *E. coli* MG1655 based on significant depletion of Tn5 insertions (log_2_FC ≤ −2 and *q* ≤ 0.01) after exposure to SMX and TMP. (**A**) Venn diagram showing total and overlapping conditionally essential genes under 1/2 MIC of TMP and SMX; (**B**) conditionally essential genes under 1/2 and 1/4 MICs of SMX; (**C**) conditionally essential genes under 1/2 and 1/4 MICs of TMP; (**D**) functional classification of conditionally essential genes for growth under SMX treatment; (**E**) functional classification of conditionally essential genes for growth under TMP treatment. Venn diagrams were generated in PowerPoint, and the bar graph was created using GraphPad Prism 10.3.0.

Comparative TraDIS-bioinformatics analysis between the control and those exposed to SMX and TMP identified 36 and 89 genes as conditionally essential, when libraries were exposed to 1/2 MIC of SMX and TMP, respectively, with 10 genes overlapping between the two conditions (Fig. 2A and Tables 1 and 2). After exposure to 1/4 MIC of SMX, five genes (*fadR, tpiA, wzxE, nlpI,* and *degP*) were identified as conditionally essential, whereas two genes (*surA* and *mtn*) were identified as conditionally essential under exposure to 1/4 MIC of TMP (Fig. 2B and C). For both drugs, all genes identified as conditionally essential under 1/4 MIC were also conditionally essential under 1/2 MIC of the corresponding antimicrobial (Fig. 2B and C). Complete lists of gene depletions after growth in the presence of SMX and TMP are shown in Table S5A through F.

**TABLE 1:** List of top 30 conditionally essential genes in MG1655_pACYC_*Sul2*(SULFA^R^) under 1/2 MIC (1,024 mg/L) of SMX

Gene_name	Function	log_2_FC	*q*-value
*wzxE^a^*	Lipid IIIECA flippase	−6.19	8.48 × 10^−308^
*yajC*	Sec translocon accessory complex subunit YajC	−6.15	2.38 × 10^−04^
*hns*	DNA-binding transcriptional dual regulator H-NS	−5.94	3.95 × 10^−127^
*fadR^a^*	DNA-binding transcriptional dual regulator FadR	−5.65	2.60 × 10^−92^
*apaH^a^^,^^b^*	Diadenosine tetraphosphatase	−5.50	1.64 × 10^−193^
*clsA*	Cardiolipin synthase A	−5.31	1.51 × 10^−164^
*rpsR*	30S ribosomal subunit protein S18	−4.35	1.55 × 10^−08^
*ftsN*	Cell division protein FtsN	−3.91	1.69 × 10^−200^
*clpP*	ATP-dependent Clp protease proteolytic subunit	−3.61	1.78 × 10^−45^
*dgkA*	Diacylglycerol kinase	−3.52	4.15 × 10^−69^
*nlpI^a^^,b^*	Lipoprotein NlpI	−3.44	2.16 × 10^−17^
*degP^a^^,b^*	Periplasmic serine endoprotease DegP	−3.34	1.58 × 10^−307^
*pcnB^b^*	Poly(A) polymerase I	−3.29	1.83 × 10^−252^
*tpiA^a^^,b^*	Triose-phosphate isomerase	−3.24	3.89 × 10^−05^
*cpxR*	DNA-binding transcriptional dual regulator CpxR	−3.21	6.11 × 10^−152^
*metJ*	DNA-binding transcriptional repressor MetJ	−3.21	1.82 × 10^−07^
*dksA*	RNA polymerase-binding transcription factor DksA	−3.13	5.54 × 10^−07^
*topA*	DNA topoisomerase 1	−3.03	3.66 × 10^−12^
*ylcJ*	Protein YlcJ	−2.98	3.94 × 10^−06^
*clpX*	ATP-dependent Clp protease ATP-binding subunit ClpX	−2.72	2.37 × 10^−48^
*surA* ^*a*^ ^*,* *b*^	Chaperone SurA	−2.68	7.05 × 10^−12^
*mgrB*	PhoQ kinase inhibitor	−2.55	2.19 × 10^−15^
*hslV*	Peptidase component of the HslVU protease	−2.45	1.76 × 10^−171^
*dnaJ*	Chaperone protein DnaJ	−2.43	1.01 × 10^−40^
*pstA*	Phosphate ABC transporter membrane subunit PstA	−2.42	6.13 × 10^−34^
*secG*	Sec translocon subunit SecG	−2.41	2.23 × 10^−46^
*pal^b^*	Peptidoglycan-associated outer membrane lipoprotein Pal	−2.38	4.14 × 10^−06^
*pstC^b^*	Phosphate ABC transporter membrane subunit PstC	−2.37	5.86 × 10^−21^
*hslU*	ATPase component of the HslVU protease	−2.36	3.44 × 10^−283^
*secM*	SecA translation regulator	−2.31	2.50 × 10^−08^

^*a*^ Genes selected for validation.

^*b*^ Genes shared between 1/2 MIC of SMX and 1/2 MIC TMP exposure.

**TABLE 2:** List of top 30 conditionally essential genes in MG1655_pACYC_*dfrA1*(TMP^R^) under 1/2 MIC (1,500 mg/L) of TMP

Gene_name	Function	log_2_FC	*q*-value
*degP^a^^,b^*	Periplasmic serine endoprotease DegP	−5.32	8.48 × 10^−308^
*dam*	DNA adenine methyltransferase	−4.43	2.38 × 10^−04^
*prc^a^*	Tail-specific protease	−4.25	3.95 × 10^−127^
*surA^a^^,b^*	Chaperone SurA	−4.17	2.60 × 10^−92^
*bamB*	Outer membrane protein assembly factor BamB	−3.96	1.64 × 10^−193^
*tpiA^a^^,b^*	Triose-phosphate isomerase	−3.75	1.51 × 10^−164^
*pstB*	Phosphate ABC transporter ATP-binding subunit	−3.71	1.55 × 10^−08^
*rfaD*	ADP-L-glycero-D-mannoheptose 6-epimerase	−3.68	1.69 × 10^−200^
*lipA*	Lipoyl synthase	−3.53	1.78 × 10^−45^
*gmhA*	D-sedoheptulose 7-phosphate isomerase	−3.38	4.15 × 10^−69^
*qseC*	Sensor histidine kinase QseC	−3.37	2.16 × 10^−17^
*cra*	DNA-binding transcriptional dual regulator Cra	−3.32	1.58 × 10^−307^
*ubiI*	2-octaprenylphenol 6-hydroxylase	−3.31	1.83 × 10^−252^
*crr*	Enzyme IIA(Glc)	−3.29	3.89 × 10^−05^
*lapA*	Lipopolysaccharide assembly protein A	−3.29	6.11 × 10^−152^
*arcA*	DNA-binding transcriptional dual regulator ArcA	−3.27	1.82 × 10^−07^
*aceE*	Pyruvate dehydrogenase E1 component	−3.20	5.54 × 10^−07^
*rppH*	RNA pyrophosphohydrolase	−3.18	3.66 × 10^−12^
*bamE*	Outer membrane protein assembly factor BamE	−3.16	3.94 × 10^−06^
*rpsP*	30S ribosomal subunit protein S16	−3.13	2.37 × 10^−48^
*nuoK*	NADH:quinone oxidoreductase subunit K	−3.12	7.05 × 10^−12^
*apaH^a^^,b^*	Diadenosine tetraphosphatase	−3.12	2.19 × 10^−15^
*nlpI^a^^,b^*	Lipoprotein NlpI	−3.05	1.76 × 10^−171^
*yciB*	Inner membrane protein	−3.03	1.01 × 10^−40^
*uof*	RyhB-regulated fur leader peptide	−2.99	6.13 × 10^−34^
*yabI*	DedA family protein YabI	−2.96	2.23 × 10^−46^
*ompA*	Outer membrane protein A	−2.93	4.14 × 10^−06^
*fur*	DNA-binding transcriptional dual regulator Fur	−2.88	5.86 × 10^−21^
*yqjA*	DedA family protein YqjA	−2.84	3.44 × 10^−283^
*nfuA*	Iron-sulfur cluster carrier protein NfuA	−2.82	2.50 × 10^−08^

^*a*^ Genes selected for validation.

^*b*^ Genes shared between 1/2 MIC of TMP and 1/2 MIC of SMX exposure.

Functional classification based on Gene Ontology (GO) terms and Kyoto Encyclopedia of Genes and Genomes (KEGG) pathways revealed that genes identified as conditionally essential were involved in key cellular mechanisms (Fig. 2D and E; Table S11). For SMX stress, these included genes related to lipopolysaccharide (LPS) biosynthesis and cell envelope integrity (*waaO, wzxE, pal, fadR, nlpI, ompA, dgkA, yajC, clsA, dsbA*, *rffC, secG,* and *secM*), stress response and protein quality control (*cpxR, clpP, clpX, phoP, degP, dnaJ, surA, hslU, hslV*, and *mgrB*), cell division and nucleotide metabolism (e.g., *zapA, ftsN,* and *apaH*), transcriptional regulation (*hns, dksA,* and *topA*), central metabolic pathways (*tpiA* and *metJ*), and plasmid copy number and translation (*pcnB* and *rpsR*). Additionally, genes involved in phosphate transport (*pstA* and *pstC*) were also classified as conditionally essential. Enrichment analysis of these genes revealed enrichment for two KEGG pathways, including protein export and the cationic antimicrobial peptide pathway. Moreover, several GO terms were enriched, primarily in the biological process category (e.g., protein denaturation and proteolysis involved in protein catabolic processes) and the cellular component category (e.g., Clp complex and HslUV protease complex) (Table S12 and S13).

The genes identified as conditionally essential for growth under 1/2 MIC of TMP were associated with envelope integrity, outer membrane assembly, and stress response (e.g., *bamB, bamE, nlpI, ompA, pal, tolA, tolB, tolQ, mlaA, mlaC, mlaE, mlaF, degP, prc,* and *surA*), LPS and enterobacterial common antigen (ECA) biosynthesis and assembly (e.g., *waaB, waaC, waaF, waaP, waaO, gmhA, hldE, wecB, wecC, lpxT, rfaD,* and *rfe*), respiratory chain components and energy production, mainly subunits of the NADH:ubiquinone oxidoreductase complex I (*nuoB–N, ndh,* and *atpA*), and central carbon and core metabolism (e.g., *aceE, aceF, icd, ackA, tpiA,* and *mtn*). Genes relevant to transcriptional regulation and signaling, such as *arcA, arcB, cra, crp, qseC, rpoS,* and *fur*, also appeared as conditionally essential, suggesting comprehensive transcriptional remodeling. DNA replication, repair, and modification genes (*dam* and *priA*) were also classified as conditionally essential under TMP stress.

Enrichment analysis of the 89 TMP-responsive genes revealed significant functional enrichment in four KEGG pathways involved in LPS biosynthesis, oxidative phosphorylation, general metabolic pathways, and amino/nucleotide sugar metabolism, highlighting envelope and metabolic-related stress responses. Gene Ontology enrichment analysis identified 26 biological processes that supported roles primarily in aerobic respiration, LPS and lipid metabolism, membrane organization, and the biosynthesis of carbohydrate derivatives. Twelve GO terms were enriched in the cellular component category, particularly pointing toward localization within membrane protein complexes, the plasma membrane, the cell periphery, the NADH dehydrogenase complex, and transmembrane transporter systems. In the category of molecular functions, six GO terms were enriched, predominantly focusing on oxidoreductase activity, active transmembrane transport, and NADH dehydrogenase (quinone/ubiquinone) activity, indicating altered membrane dynamics and redox metabolism in response to TMP stress (Table S14 and S15).

### Selection of conditionally essential genes for validation of TraDIS results

To validate the TraDIS findings, 10 conditionally essential genes were selected. Among these, six genes (*apaH, degP, nlpI, surA, waaO*, and *tpiA*) were common to both drug conditions, while two genes (*prc* and *mtn*) were unique to the TMP-treated library, and two genes (*wzxE* and *fadR*) to the SMX library. The genes represented diverse functions; *prc* and *nlpI* are associated with peptidoglycan (PG) metabolism, *fadR* and *waaO* participate in fatty acid metabolism and LPS biosynthesis, *degP* and *surA* are chaperones involved in protein degradation and folding, *tpiA* performs a key role in glycolysis, and *apaH* is indirectly linked to nucleotide metabolism. The *wzxE* and *mtn* are part of the ECA biosynthesis and methionine salvage pathway, respectively.

Each gene was individually deleted in *E. coli* MG1655. To investigate the impact of the lack of these genes in strains with different SMX and TMP resistance genes, plasmids encoding *sul1, sul2*, or *sul3* for SMX resistance, and *dfrA1*, *dfrA5*, or *dfrA12* for TMP resistance were individually transformed into each of the 10 deletion mutants.

### Validation of selected conditionally essential genes through growth curve analysis

In control (antibiotic-free conditions), SMX-resistant parent strains carrying *sul1, sul2*, or *sul3* genes and their respective knockout mutants showed very similar growth patterns, with only slightly longer lag phases or slower growth rates of the mutants compared to the parent strains. Notable exceptions were the *surA* mutants, which showed 3–4 h longer lag phases in the *sul1* and *sul3* backgrounds, and the Δ*prc* mutants, which never reached the same OD level as the parent strain when they leveled out (Fig. S4 to S6).

In the presence of 1/2 MIC of SMX, mutants generally exhibited considerably delayed growth or complete inhibition compared to their parent resistant strains. For the *sul1*-carrying strains, Δ*degP,* Δ*wzxE,* Δ*apaH, and* Δ*fadR* mutants exhibited extended lag phases, ranging from 4 to 10 h, compared to the parent resistant strain, while their subsequent growth appeared comparable to that of the parent strain. In contrast, Δ*tpiA,* Δ*nlpI,* and Δ*prc* mutants failed to grow*,* while Δ*waaO and* Δ*surA* showed nearly no growth. The *mtn* mutant appeared unaffected (Fig. S7). Overall, although some mutants eventually entered exponential growth, none attained the OD reached by the parent resistant strain during the 24-h measurement period.

**Fig 3:**
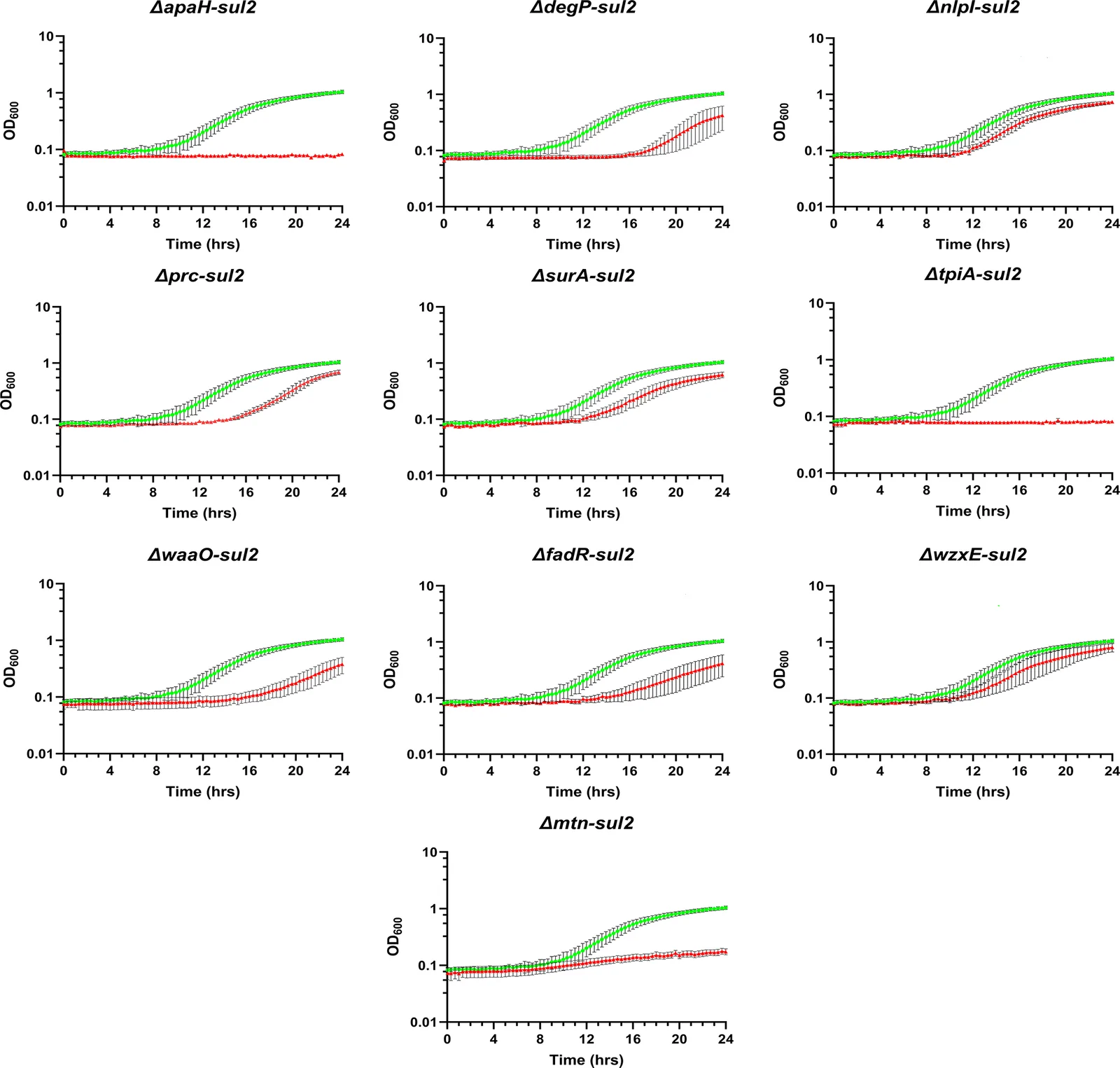
Growth curves of mutants with *sul2* background in the presence of SMX. Growth kinetics of sulfa-resistant parent strain MG1655/pACYC_*sul2*(SULFA^R^) (green line) compared to its respective 10 deletion mutants (red line) in LB medium containing 1,024 µg/mL SMX. Curves are generated by GraphPad Prism 10.3.0 and represent the mean ± standard deviation of three independent biological replicates, each with three technical replicates.

In *sul2*-carrying strains in the presence of 1/2 MIC of SMX, the Δ*wzxE* and Δ*nlpI* mutants exhibited only a slight growth defect, while more pronounced growth impairment was observed in the Δ*surA*, Δ*fadR*, Δ*degP*, Δ*prc*, and Δ*waaO* mutants, mainly associated with an extended lag phase ranging from 4 to 8 h longer than that of the parent resistant strain. No or only very limited growth was detected for the Δ*apaH*, Δ*tpiA*, and Δ*mtn* mutants (Fig. 3). Among the mutants that grew, all reached lower OD than the parent strain in 24 h of incubation, except Δ*wzxE*, which showed comparable OD. The *sul3*-carrying mutants also displayed pronounced growth defects. Δ*apaH*, Δ*tpiA*, Δ*mtn*, Δ*fadR,* and Δ*prc* mutants failed to grow or showed only minimal growth, while Δ*waaO,* Δ*wzxE,* Δ*nlpI,* and Δ*surA* showed a prolonged lag phase of 4–9 h compared to the parent resistant strain and exhibited growth comparable to the parent strain once in the growth phase. The Δ*degP* mutant grew almost similarly to the parent strain (Fig. S8). Mutants that entered the growth phase did not reach the OD level of the parent strain within 24 h, except for Δ*wzxE* and Δ*degP*.

Under antibiotic-free (control) conditions, some knockout mutants exhibited growth patterns comparable to their TMP-resistant parent strains harboring *dfrA1*, *dfrA5*, or *dfrA12* genes. However, Δ*fadR,* Δ*mtn,* Δ*tpiA*, and Δ*wzxE* mutants showed lag phases extended by 1–2 h, and Δ*surA* exhibited a 2–3 h longer lag phase compared to the parent strain (Fig. S9 to S11).

**Fig 4:**
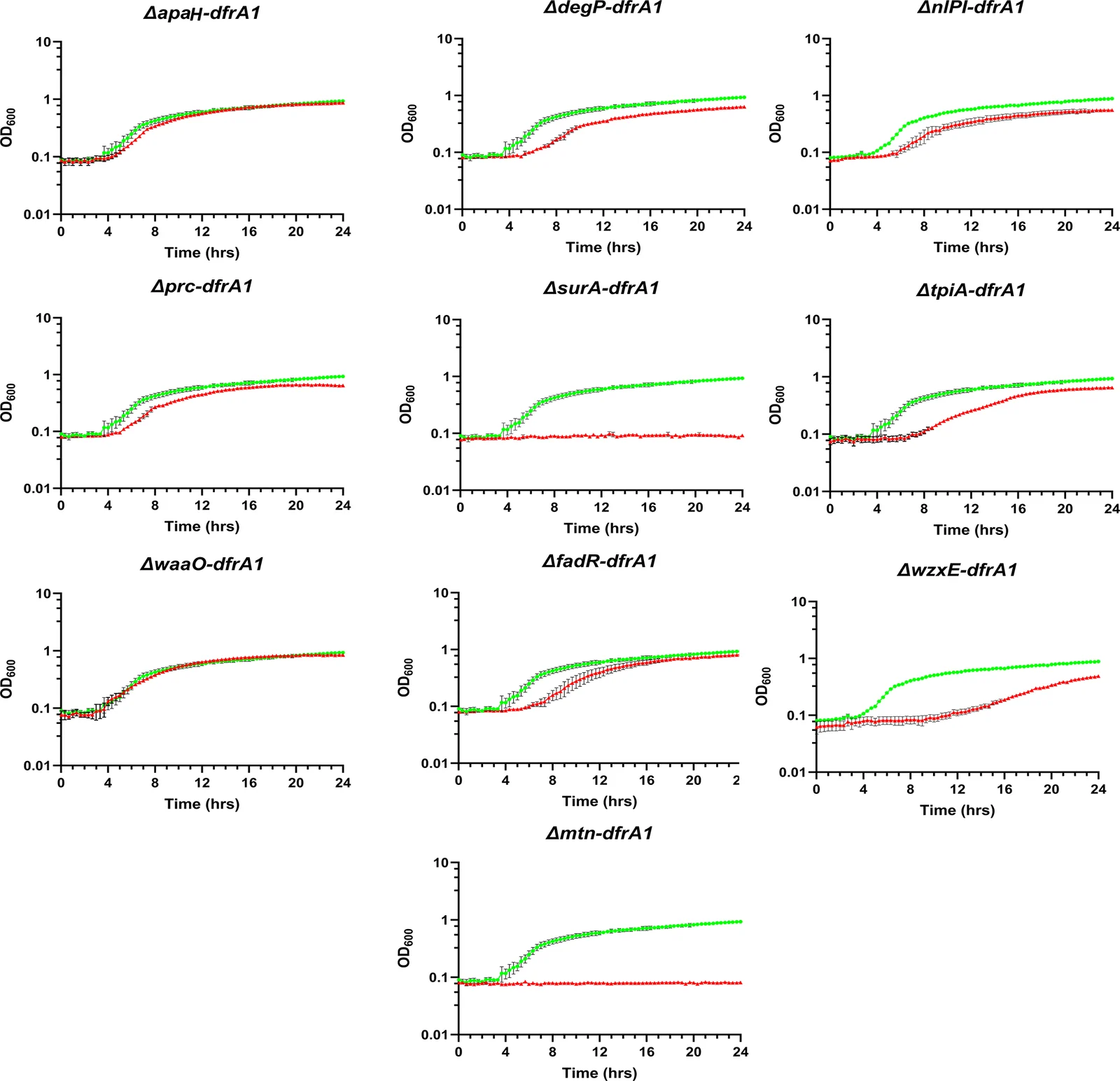
Growth curves of mutants with *dfrA1* background in the presence of TMP. Growth kinetics of TMP-resistant parent strain MG1655/pACYC_*dfrA1*(TMP^R^) (green line) compared to its respective 10 deletion mutants (red line) in LB medium containing 1,500 mg/L TMP. Curves are generated by GraphPad Prism 10.3.0 and represent the mean ± standard deviation of three independent biological replicates, each with three technical replicates.

Under 1/2 MIC TMP exposure, knockout mutants showed varying growth responses; however, these responses were almost consistent across the three TMP resistance genes, *dfrA1*, *dfrA5*, or *dfrA12*. The Δ*mtn,* Δ*surA,* Δ*wzxE,* and Δ*fadR* mutants showed no or very limited growth, except that the Δ*fadR* mutants grew with an extended lag phase in the *dfrA1* gene background. The Δ*degP,* Δ*nlpI,* Δ*prc*, and Δ*tpiA* mutants exhibited a prolonged lag phase of 3–4 h, whereas the Δ*waaO* and Δ*apaH* mutations did not result in growth changes compared to the resistant parent strains (Fig. 4; Fig. S12 and S13).

### Validation of TraDIS data through MIC testing

A total of 60 mutant strains (30 sulfa-resistant and 30 TMP-resistant ones) were tested, derived from 10 individual *E. coli* MG1655 gene knockout mutants (*degP, nlpI, prc, fadR, wzxE, surA, apaH, mtn, tpiA,* and *waaO*) carrying either sulfa-resistance genes (*sul1, sul2*, or *sul3*) or trimethoprim-resistance genes (*dfrA1, dfrA5*, or *dfrA12*). Under SMX exposure, all mutants across *sul1, sul2,* and *sul3* backgrounds exhibited a twofold reduction in MICs compared to their respective sulfa-resistant parent strains, indicating increased susceptibility (Tables S16 to S18).

Similarly, TMP exposure revealed that deletion of the same set of genes in strains carrying *dfrA1*, *dfrA5*, or *dfrA12* resulted in a uniform reduction in MIC for specific gene knockouts. Particularly, Δ*mtn* mutants demonstrated the highest increase in susceptibility, with up to an eightfold MIC reduction, while Δ*surA* mutants showed a fourfold decrease in MIC across *dfrA1, dfrA5,* or *dfrA12* backgrounds. Δ*degP,* Δ*nlpI,* Δ*prc,* Δ*fadR,* Δ*wzxE, and* Δ*tpiA* mutants only exhibited a twofold reduction in MIC. Conversely, and in agreement with the results of growth assays, the MICs for the Δ*apaH* and Δ*waaO* mutants were the same as those of the resistant parent strains (Tables S19 to S21).

### Validation of TraDIS data through competition growth assay

Following monoculture growth curve analysis and MIC testing, we conducted a competition experiment to evaluate the relative fitness of selected SMX- and TMP-resistant knockout mutants compared to their resistant parent strains under control and antibiotic exposure conditions. Mutants chosen for the competition assay were those that showed attenuated growth with extended lag phases in individual growth curves; mutants with total growth arrest were not included.

**Fig 5:**
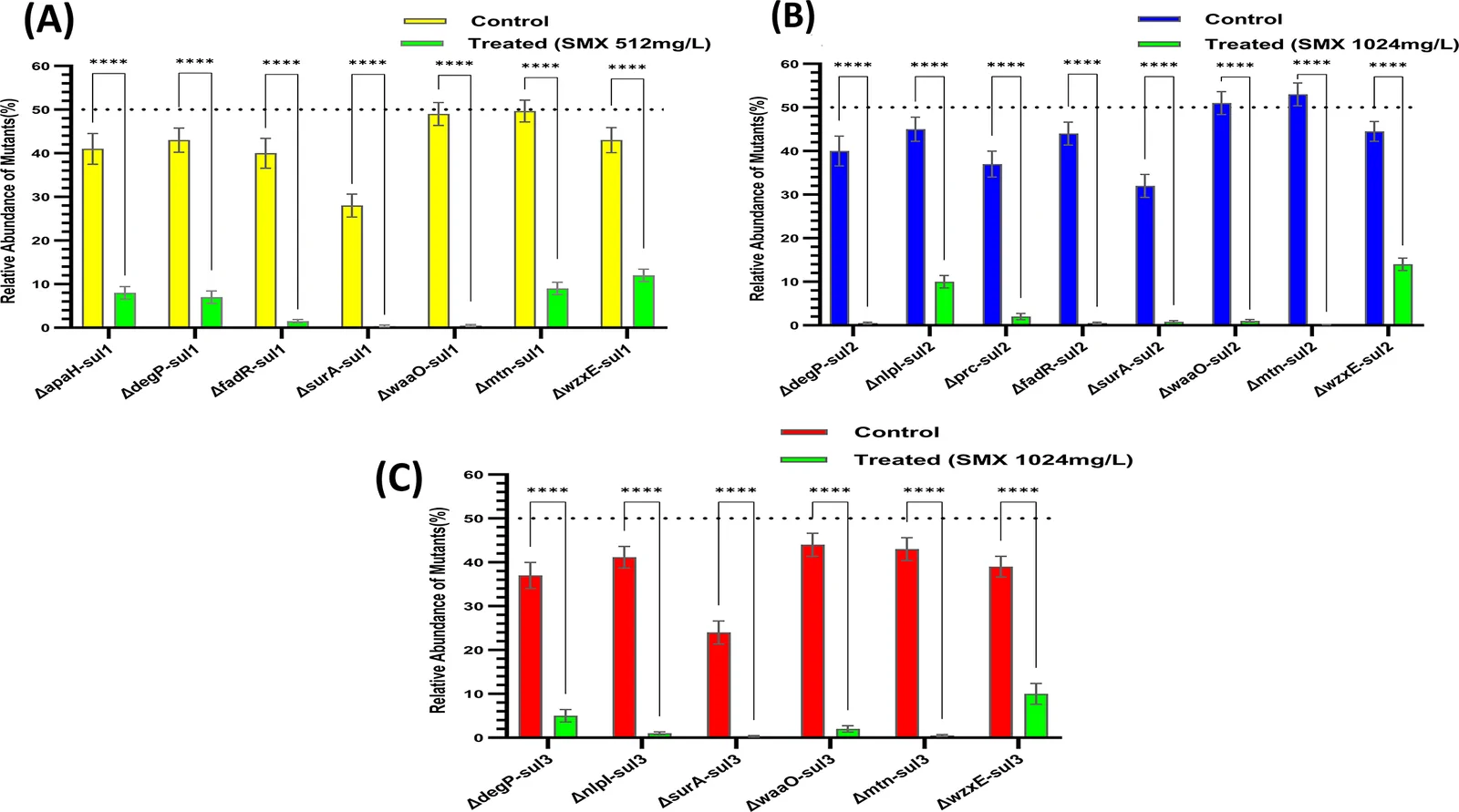
Competition between sulfonamide-resistant parent *E. coli* strains carrying (**A**) *sul1*, (**B**) *sul2*, or (**C**) *sul3* and their respective deletion mutants, with and without SMX (1,024 µg/mL). Relative abundance (%) of KO mutants after 24 h of co-culture is shown. Yellow, blue, and red bars indicate the relative abundance of KO mutants in control conditions for *sul1*, *sul2*, and *sul3* backgrounds, respectively; green indicates the relative abundance under SMX exposure across all backgrounds. Competitions were initiated with equal concentrations of KO mutants and parent strains. Relative abundance was determined by CFU counts after 24 h of incubation. The dotted line denotes 50% relative abundance. Data represent mean ± SD from two biological and three technical replicates; ****P < 0.0001 indicates a significant difference. Graphs were created with GraphPad Prism 10.3.0.

In the control conditions (without antibiotics), the relative abundance of sulfa-resistant knockout mutants (Δ*apaH,* Δ*degP,* Δ*nlpI,* Δ*prc,* Δ*fadR*, Δ*surA,* Δ*waaO,* Δ*wzxE*, and Δ*mtn*) ranged from 24% to 53%, with most falling between 40% and 50% (Δ*apaH,* Δ*degP,* Δ*nlpI,* Δ*fadR,* Δ*waaO,* Δ*wzxE*, and Δ*mtn*). However, when exposed to 1/2 MIC of SMX, a significant decrease (ranging from <1% to 14%, ****P < 0.0001 compared to range without antibiotics) in the relative abundance of knockout mutants was observed (Fig. 5). Mutants with the *sul3* background showed a slightly less competitive advantage than those with *sul1* or *sul2* under control conditions, and for certain knockouts (Δ*nlpI* and Δ*wzxE),* also under SMX treatment.

**Fig 6:**
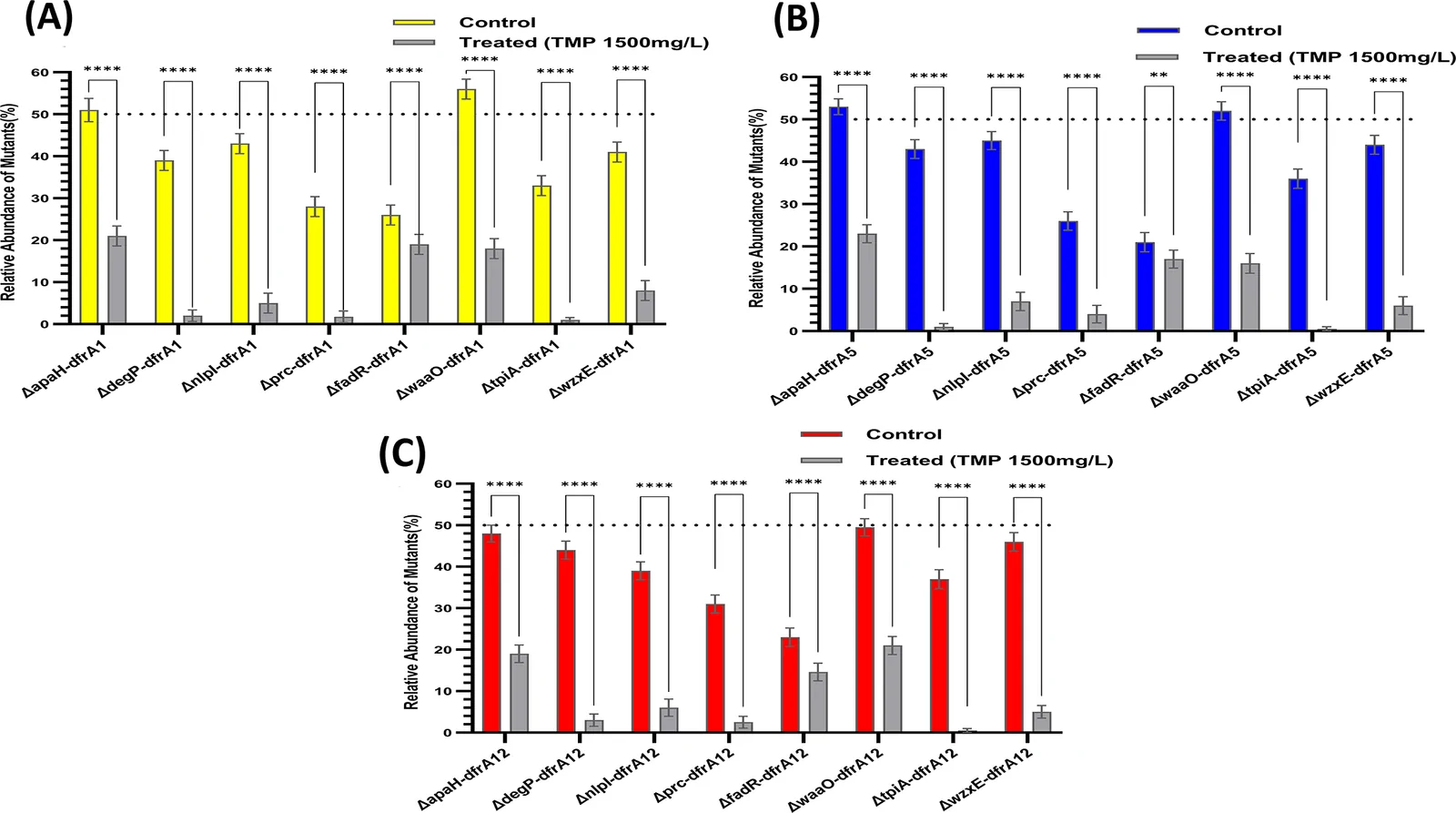
Competition between trimethoprim-resistant parent *E. coli* strains carrying (**A**) *dfrA1*, (**B**) *dfrA5*, or (**C**) *dfrA12* and their respective deletion mutants, with and without TMP (1,500 µg/mL). Relative abundance (%) of KO mutants after 24 h of co-culture is shown. Yellow, blue, and red bars represent the relative abundance of KO mutants in control conditions for *dfrA1, dfrA5*, and *dfrA12* backgrounds, respectively; gray indicates the relative abundance under TMP exposure across all backgrounds. Competitions were initiated with equal concentrations of KO mutants and parent strains. Relative abundance was determined by CFU counts after 24 h of incubation. The dotted line denotes 50% relative abundance. Data represent mean ± SD from two biological and three technical replicates; ****P < 0.0001; **P < 0.01 indicates a significant difference. Graphs were created with GraphPad Prism 10.3.0.

Competition among TMP-resistant knockout mutants (Δ*apaH,* Δ*degP,* Δ*nlpI,* Δ*prc,* Δ*fadR,* Δ*waaO,* Δ*tpiA,* and Δ*wzxE*) and their *TMP-*resistant parent strains revealed relative abundances ranging from 21% to 56%, with the majority between 40% and 50% (Δ*apaH,* Δ*degP,* Δ*nlpI,* Δ*waaO,* and Δ*wzxE*) in antibiotic-free conditions. A sharp decline in their abundance (ranging between <1% and 23%, ****P < 0.0001 compared to range under antibiotic-free conditions) was observed upon exposure to 1/2 MIC of TMP (Fig. 6), regardless of the TMP-resistance gene variant (*dfrA1, dfrA5,* and *dfrA12*).

### Differential gene essentiality under antifolate stress in resistant versus susceptible strains

To assess how plasmid-mediated resistance alters antifolate responses, we reanalyzed the TraDIS data set from Turner et al. (40), who investigated the susceptible *E. coli* BW25113 strain exposed to 2×, 1×, 1/2×, and 1/4× MIC of SMX and TMP. Using comparable thresholds (log₂FC ≤ −2 and *q* ≤ 0.01), no overlap was found for TMP-associated genes, whereas five genes, *wzxE, hns, fadR, nlpI,* and *prc*, were shared between the SMX conditions in the two studies.

### Homology analysis

Homology analysis was performed on all 10 candidate genes that were validated as conditionally essential. This identified two conditionally essential *E. coli* genes, *tpiA* and *degP*, as conserved across humans and diverse animal species. TpiA showed 98% similarity to human TPI1 and 96%–98% to its orthologs in cat, dog, pig, cow, horse, sheep, goat, chicken, rabbit, and buffalo. DegP shared 50%–54% similarity with human HTRA serine peptidases and 36%–52% across the same animals. The remaining eight conditionally essential genes (including *mtn, apaH, surA, waaO, wzxE, prc, fadR*, and *nlpI*) showed no detectable homology to human or animal proteins. All analyzed genes were conserved throughout *Enterobacteriaceae*, including different genera (Table S22).

## DISCUSSION

Due to the rise in antimicrobial resistance and the slow rate of new antimicrobial discovery, there is an urgent need to explore alternative approaches to combat resistant pathogens. Resistance in *E. coli* to commonly used antibiotics, such as sulfamethoxazole and trimethoprim, is becoming increasingly prevalent in clinical isolates, limiting treatment options (41). To tackle this challenge, a possible approach is to make the reuse of these older antibiotics possible by identifying helper drugs. This strategy, which can help to re-sensitize resistant *E. coli* strains to SMX and TMP by targeting gene products that are conditionally essential in resistant bacteria under antibiotic exposure, is the subject of the current study.

TraDIS has emerged as a robust, high-throughput technique for identifying non-essential genes that become conditionally essential for the fitness and survival of bacteria under various stressful environments, including antibiotic exposure (30). Studies have applied this approach to find conditionally essential genes important for the growth of *E. coli and K. pneumoniae* in the presence of aminoglycosides, β-lactams, chloramphenicol, colistin, ciprofloxacin, and macrolides (17, 18, 20, 21, 23). Based on this, our study employed the TraDIS approach to identify conditionally essential genes in SUL- and TMP-resistant *E. coli* exposed to SMX and TMP.

TraDIS analysis classified 335 genes as essential for the growth of *E. coli*. These genes were common to SMX- and TMP-resistant *E. coli* libraries and represented conserved core functions, typically essential for bacterial viability. The number of essential genes is consistent with earlier predictions of the number of essential genes in *E. coli* measured by the TraDIS technique (18, 21), supporting the saturation of our transposon mutant libraries and their suitability for downstream functional genomics investigations.

A total of 5 and 36 genes were found to be conditionally essential under 1/4 MIC and 1/2 MIC of SMX, respectively, while 2 and 89 conditionally essential genes were identified under 1/4 MIC and 1/2 MIC of TMP. Only 10 of the conditionally essential genes overlapped between the two antimicrobial challenges, suggesting that although the resistance genes *sul2* and *dfrA1* confer resistance to SMX and TMP through inhibition of the folate metabolic pathway, the secondary genes needed to support resistance expression in *E. coli* are largely different. In both libraries, genes identified at the lower concentration (1/4 MIC) were also conditionally essential at the higher concentration (1/2 MIC), indicating that these genes likely encode the factors most crucial for growth in the presence of these antimicrobials. Conversely, the conditional essentialities observed at 1/2 MIC were mostly lost when the concentration was lowered.

Under SMX stress, *E. coli* growth depended on diverse mechanisms involving 26 unique conditionally essential genes, some of which overlapped with dependencies identified for other classes of antimicrobials. Thus, similar to aminoglycoside responses (21), cell envelope integrity genes, cell division genes, and various stress response systems, including chaperones and regulators, were classified as conditionally essential. The latter group was also identified in β-lactam-resistant *E. coli* under β-lactam stress (18). Another group of genes was classified as transcriptional regulators. These genes have previously been identified as conditionally essential during the growth of resistant *E. coli* in the presence of both aminoglycosides and macrolides (20, 21). Together, these observations indicate that *E. coli* employs similar adaptive strategies during growth in the presence of different antimicrobial classes.

In response to TMP exposure, TraDIS analysis identified 79 genes that were uniquely conditionally essential under TMP stress, highlighting genetic determinants that contribute to bacterial fitness during antifolate exposure. Among these, 12 genes from the *nuo* operon suggested a key role for NADH dehydrogenase I under TMP stress. A previous study has reported *nuo* upregulation during meropenem exposure in *E. coli* (42), once again suggesting shared responses across different classes of antimicrobials. Several genes involved in envelope biogenesis and stability, including eight genes directly involved in LPS biosynthesis, three O-antigen biosynthesis genes, and four Tol-Pal system genes (*tolABQ* and *pal*), revealed that compromised outer membrane integrity is critical for *E. coli* under TMP stress. The identification of the *wec* genes as a fitness determinant under aminoglycoside stress (21) and the Tol-Pal system under macrolide stress (20) suggests that these envelope-associated pathways may serve as common vulnerability points across multiple antibiotic classes, possibly opening for helper drugs against different drugs. Outer and inner membrane-associated genes were further associated with TMP stress responses, suggesting that TMP may indirectly trigger membrane stress through metabolic disruption. Among these, *bamB/E* genes were previously shown to be conditionally essential under CHL and macrolide stress in resistant *E. coli* (17, 20). The list of conditionally essential genes further included genes involved in central metabolism, phosphate uptake, DNA repair and replication, and redox regulation, further supporting the notion that multiple physiological systems become conditionally essential under TMP exposure.

Only five genes overlapped between the SMX-treated susceptible strain from Turner et al. (40) and the resistant strains in the current study, and no overlap was observed for TMP-associated genes. This limited overlap indicates that plasmid-mediated resistance reshapes the bacterial response to antifolate stress. While susceptible strains rely on a specific set of stress adaptation pathways, resistant strains appear to engage alternative mechanisms, particularly those linked to envelope integrity, protein quality control, stress response, and energy metabolism. These findings emphasize the impact of resistance determinants on cellular physiology and the importance of carrying out studies with resistant bacteria to identify genetic vulnerabilities and potential therapeutic targets.

To validate the TraDIS predictions, we selected 10 conditionally essential genes for knockout. This included all genes identified at 1/4 MIC for both antibiotics. Additionally, two genes, *apaH* and *waaO*, shared between the two libraries at 1/2 MIC, and *prc*, unique to the TMP 1/2 MIC condition, were selected. These three genes represent different pathways, i.e., diadenosine tetraphosphate (Ap4A) hydrolysis (*apaH*), LPS biosynthesis (*waaO*), and peptidoglycan remodeling (*prc*). In all, the selection included two SMX-unique genes (*fadR* and *wzxE*), two TMP-unique genes (*mtn* and *prc*), and six genes shared between the two drugs (*degP, nlpI, tpiA, apaH, waaO,* and *surA*). To investigate whether results obtained with *E. coli* strains resistant to either *sul2* or *dfrA1* were valid for a broader selection of resistance genes against sulfa and TMP, the validation included *E. coli* strains expressing resistance encoded by *sul1, sul3, dfrA5,* and *dfrA12*.

Under SMX stress, some knockout mutants exhibited distinct growth patterns across *sul1–3* backgrounds. Disruptions of *apaH* resulted in no growth in the *sul2* and *sul3* backgrounds, while the mutant grew in the *sul1* background with a delayed lag phase. Deletion of *prc* abolished growth in *sul1* and *sul3*, while growth resumed after an extended lag phase in *sul2* backgrounds. The *nlpI* and *waaO* knockout mutants exhibited little or no growth in the *sul1* background but grew in the *sul2* and *sul3* backgrounds after a long lag phase. Together, these results show that the conditional essentiality varies with the different resistant genes. This underscores the importance of testing a wider range of genetic resistance mechanisms in the search for suitable helper drug targets. Knockout of *tpiA* consistently abolished growth of the mutants in the presence of 1/2 MIC of SMX in all *sul* backgrounds, indicating its fundamental role in SMX tolerance. The *surA, fadR,* and *wzxE* deletions also resulted in pronounced fitness defects, primarily associated with prolonged lag phases. The *sul3* variant generally had a higher fitness cost compared to its *sul2* counterparts. This observation supports a previous *in vitro* study, which found that *sul3* exhibited a higher fitness cost than the *sul1* and *sul2* genes, potentially linked to the altered expression of energy-relevant ABC transporters such as UgpC, RbsA, and GsiA (43). The *mtn* gene appeared to make a special case. In the *sul2 and sul3* backgrounds, no or very limited growth was observed in the presence of SMX when this gene was removed. In contrast, knockout did not seem to make a difference in the *sul1* background. The reason for this observation is unclear, and further studies are warranted to gain a deeper understanding. Taken together, the results of the SMX validation suggest that different variants of closely related resistance genes, such as the *sul* genes, are likely to have similar secondary resistomes. However, they can occasionally exhibit distinct physiological responses in the presence of antibiotics.

The most promising candidate genes for disrupting the growth of resistant bacteria were selected for validation in competition assays. Contrary to growth assay, where strains were grown individually, competition assays showed clear outcompeting of gene mutants by the parent resistant strain, as the proportion of mutants dropped sharply from 30%–50% to <1%–14% under SMX stress. This difference compared to single-strain growth analyses probably reflects that the mutants were initially selected as those that failed to compete in TraDIS, where each Tn mutant was competing against several hundred thousand other mutants for growth. As a final validation, MIC testing confirmed the predictions from TraDIS in that all knockout mutants showed a twofold reduction from 1,024 to 512 µg/mL in the *sul1* backgrounds and from 2,048 to 1,024 µg/mL in the *sul2* and *sul3* backgrounds, documenting increased susceptibility of the mutants.

During TMP exposure, we also observed consistent fitness loss across all three *dfrA* variants. Deletion mutants of *surA* and *mtn* completely stopped growth and showed a fourfold decrease in MIC for Δ*surA* and an eightfold decrease for Δ*mtn*, emphasizing the crucial roles these mutations play under DHFR inhibition. Both here and for SMX challenge, it should be noted that the *surA* mutation also caused growth decrease during growth in the absence of antibiotic, making it difficult to evaluate how big a proportion of the growth inhibition was caused by antimicrobial challenge. However, competition experiments showed a significant additional effect of the knockout when antimicrobials were added, and we think the gene product may be a candidate for helper drug target. Mutants lacking *degP, nlpI, prc, fadR, tpiA*, and *wzxE* displayed reduced growth, longer lag phases, twofold MIC decreases, and competitive fitness declines to <1%–23% in all three resistance-gene backgrounds. This suggests that the loss of fitness under TMP stress is primarily due to the absence of specific conditionally essential genes, rather than the specific resistance variant.

As an internal control, our testing of mutants included cross-testing, i.e., each mutant was subjected to growth under both SUL and TMP stress, irrespective of whether it had been identified as conditionally essential for only one or both antibiotics in the TraDIS analyses. Surprisingly, this revealed vulnerabilities that had not been pointed out by the TraDIS analyses. For example, *wzxE* and *fadR* were specifically conditionally essential to SMX and *mtn* and *prc* to TMP in the initial TraDIS screenings. Nevertheless, the deletion of *mtn* and *prc* effectively sensitized SMX-resistant strains to SMX, and *wzxE* deletion enhanced TMP sensitivity. This observation probably relates to the cutoff criteria, which were set to log₂FC ≤ −2 and *q* ≤ 0.01, resulting in false-negative characterization of certain genes by TraDIS. In this sense, conducting cross-drug validation studies helped us overcome the problem of false-negative prediction and identified a broader range of putative helper drug targets against SMX- and TMP-resistant bacteria.

No further studies on the mechanisms by which the validated genes affected resistance to SMX and TMP were conducted; however, previously reported studies provide clues as to how this may happen. *apaH* encodes the diadenosine tetraphosphatase responsible for clearance of the stress response signal “alarmone” diadenosine tetraphosphate in both prokaryotes and eukaryotes (44, 45). Elevated Ap4A levels caused by the loss of *apaH* activity have been reported to lead to extensive membrane disruption, increasing the effectiveness of aminoglycosides 100- to 5,000-fold (46). Although Ap4A can also inhibit IMPDH and disturb purine balance (47), this mechanism is less likely under antifolate stress, where THF depletion already limits purine synthesis. Therefore, the most plausible explanation for the conditional essentiality of *apaH* under SMX and TMP treatment is that the accumulation of Ap4A exacerbates membrane damage, thereby reducing bacterial fitness. The *tpiA* (triosephosphate isomerase) is involved in isomerization in glycolysis and gluconeogenesis. Its appearance as a conditionally essential gene under TMP and SMX stress points to a connection between central carbon metabolism and cell envelope maintenance during antibiotic challenge. Our data, consistent with reports for other antibiotics (18, 21), indicate that SMX and TMP stimulate a stress response, likely increasing the demand for glyceraldehyde-3-phosphate (G3P) to sustain membrane integrity (48). The role of *tpiA* in balancing the DHAP/G3P pool is critical, as the inadequate level of G3P could compromise envelope integrity, while surplus DHAP might lead to toxic methylglyoxal buildup (49). Certain glycolytic genes (i.e., *gapA, pgk, eno*, and *fbaA*) are generally essential in bacteria (50), while others are considered non-essential. The absence of other non-essential glycolytic genes (*pykA/F, pfkB*, and *gpmA*) in our list of conditionally essential genes reveals a unique role of *tpiA* in substrate-level regulation rather than a broader requirement of glycolysis in SMX and TMP stress.

The gene, *mtn,* encodes 5′-methylthioadenosine/S-adenosylhomocysteine nucleosidase. Its essential role in the current study probably relates to its dual role in methionine and adenine salvage and SAM recycling (51). When *de novo* purine and methionine synthesis is limited by SMX or TMP, this enzyme cleaves MTA (a polyamine byproduct) and SAH (a methylation byproduct) to regenerate adenine and homocysteine, important for nucleotide formation and SAM-dependent methylation, respectively (51). Without *mtn*, unrecycled MTA/SAH accumulates, inhibiting polyamine synthesis and methyltransferases (51).

The *surA* and *degP* (periplasmic chaperones) emerged as conditionally essential under both SMX and TMP exposure. The *surA* gene product is vital for the folding and assembly of outer membrane proteins, which are crucial for membrane integrity (52). DegP, on the other hand, helps to degrade damaged and misfolded proteins (53). Notably, these genes have also been reported as fitness determinants in *E. coli* and *P. aeruginosa* under treatment with macrolides, colistin, gentamicin, and cefotaxime (18, 20, 23). Their essentiality suggests that preserving envelope integrity is a key fitness mechanism during exposure to TMP, SMX, and other antimicrobial agents. The *nlpI* and *prc* genes were previously identified as conditionally essential for growth under macrolide exposure in resistant *E. coli* (20). Their conditional essentiality under SMX and TMP treatment underscores a central role of PG remodeling in sustaining cell envelope integrity during antibiotic stress. NlpI serves as an outer membrane adaptor that controls the activity of the PG-cleaving enzyme MepS, while also regulating its levels by engaging the protease Prc for controlled degradation (54). This balance ensures effective PG hydrolysis without compromising cell wall integrity. Under SMX and TMP exposure, where envelope stress is elevated, proper coordination of MepS activity via NlpI-Prc probably becomes crucial for maintaining the structural integrity of the cell envelope. The *wzxE* is important for ECA biosynthesis, *waaO* in LPS assembly, and *fadR* in regulating fatty acid synthesis (55–57). While this, too, underlines the critical role of membrane integrity during SMX and TMP exposure in *E. coli*, further studies are needed to determine the exact mechanism by which the product of these genes contributes to resistance to the two drugs.

Homology analysis revealed that bacterial TpiA and DegP exhibit significant similarity with their homologous proteins in humans and other animals. This may limit the potential of these proteins as a therapeutic target without further analysis of functional differences, which could lead to possible off-target toxicity. The absence of human and animal homologs for eight genes searched, particularly *mtn, apaH, surA, waaO, nlpI, prc, fadR,* and *wzxE*, highlights their potential as targets for helper drugs, particularly given their conservation across *Enterobacteriaceae* and other bacteria.

The limitations of the TraDIS approach, discussed by other authors in previous studies (16, 30, 58), are also applicable here. These include the Tn5 transposon’s tendency to target AT-rich sites, biases based on orientation, the potential to overlook essential genes involved in fitness, reduced sensitivity for detecting very short genes or sRNAs, and a tendency to overestimate the number of essential and fitness genes, and occasionally an ambiguous classification of genes. For example, in our data set, the gene *gyrB* was labeled as conditionally essential only at 1/4 MIC of TMP. *gyrB* encodes an essential DNA gyrase subunit (59), and it also appeared on the list of essential genes in the current study, in the list known as the Keio collection, and in other TraDIS studies (18, 21, 60). We considered the prediction of being conditionally essential as a likely false positive, probably caused by permissive edge insertions or noise in fitness signals. This highlights a challenge inherent to transposon-based profiling; for example, genes that are universally essential with limited terminal insertions may sometimes be marked as conditionally essential. Another weakness in the current study was that all experiments were conducted with the laboratory-adapted *E. coli* MG1655 strain, which was further grown in LB medium. This approach may not fully model clinical isolates or *in vivo* conditions. Therefore, some conditionally essential genes identified under SMX and TMP stress could differ in clinical strains or host environments, necessitating further validation across diverse strains and *in vivo* models.

In conclusion, the current study identified conditionally essential genes for growth in the presence of SMX and TMP in *E. coli* resistant to these two drug classes. The results suggest that maintaining cell envelope integrity, purine balance, and methionine salvage pathways is essential for supporting resistance toward SMX and TMP, offering new avenues to combat SMX and TMP resistance by targeting these pathways for helper drug construction.

## Data Availability

Plasmid sequences have been deposited in the UCPH ERDA Research Data Archive and are available at http://doi.org/10.17894/ucph.7457f751-c7d5-4823-a372-9d365f5b46ca. Illumina TraDIS sequencing data, metadata, supplemental tables, and supplemental figures have been deposited in the UCPH ERDA Research Data Archive and are available at http://doi.org/10.17894/ucph.5a5681c0-2b58-48d7-8c73-4d5d8ef447b5.

## SUPPLEMENTAL MATERIAL

Supplemental Tables spectrum.00603-26-s0001.xlsx Supplemental Tables S1–S22.

Supplemental Figures spectrum.00603-26-s0002.docx Supplemental Figures S1–S13.

ASM does not own the copyrights to Supplemental Material that may be linked to, or accessed through, an article. The authors have granted ASM a non-exclusive, world-wide license to publish the Supplemental Material files. Please contact the corresponding author directly for reuse.
