# Supplementary material for: Genome-wide identification of conditionally essential genes for growth in the presence of sulfamethoxazole and trimethoprim in sulfamethoxazole- and trimethoprim-resistant Escherichia coli
Source: Microbiol Spectr. 2026 Jul 29;14(9):e00603-26. doi: 10.1128/spectrum.00603-26 (PMC13532273; doi:10.1128/spectrum.00603-26)
Supplement: Supplementary file 1 [file spectrum.00603-26-s0002.docx]

**Supplementary Figures**

**Supplementary Figure S1:** SnapGene-generated maps of pACYC184-derived plasmids harboring sulfonamide resistance genes: (A) *sul1*, (B) *sul2*, and (C) *sul3*. Each construct retains the original backbone (pACYC184_ΔTet^R^ (CHL^R^) and incorporates the respective resistance gene**.**

**Supplementary Figure S2:** SnapGene-generated maps of pACYC184-derived plasmids harboring Trimethoprim resistance genes: (A) *dfrA1,* (B) *dfrA5, and* (C) *dfrA12*. Each construct retains the original backbone (*pACYC184_*Δ*TetR (CHLR)* and incorporates the respective resistance gene.

**Supplementary Figure S3:** Genome-wide mapping of Tn5 insertion sites to reference genome *E. coli* MG1655 U00096.3. The outer black ring represents the base positions, starting at the annotated origin. Sense and antisense strands are shown in green and pink, respectively. Red spikes indicate the genomic location and density of Tn5 insertions identified by TraDIS analysis. (A) SULFA-resistant input library contained 501,096 unique insertion sites (UIS). (B) TMP-resistant input library contained 462,355 UIS.

**Supplementary Figure S4:** Growth curves of mutants with *sul1* background in antibiotic-free conditions**.** Growth kinetics of sulfa-resistant parent strain *MG1655/pACYC184_sul1(SULFA^R^*) (green line) compared to its respective ten deletion mutants (red line) in LB Medium. Curves are generated by GraphPad Prism 10.3.0 and represent the mean ± standard deviation of three independent biological replicates, each with three technical replicates

**Supplementary Figure S5**: Growth curves of mutants with *sul2* background in antibiotic-free conditions. Growth kinetics of sulfa-resistant parent strain *MG1655/pACYC184_sul2(*SULFA^R^) (green line) compared to its respective ten deletion mutants (red line) in LB Medium. Curves are generated by GraphPad Prism 10.3.0 and represent the mean ± standard deviation of three independent biological replicates, each with three technical replicates.

**Supplementary Figure S6:** Growth curves of mutants with *sul3* background in antibiotic-free conditions**.** Growth kinetics of sulfa-resistant parent strain *MG1655/pACYC184_sul3(SULFA^R^*) (green line) compared to its respective ten deletion mutants (red line) in LB Medium. Curves are generated by GraphPad Prism 10.3.0 and represent the mean ± standard deviation of three independent biological replicates, each with three technical replicates.

**Supplementary Figure S7:** Growth curves of mutants with *sul1* background in the presence of SMX**.** Growth kinetics of sulfa-resistant parent strain *MG1655/pACYC184_sul1(SULFA^R^*) (green line) compared to its respective ten deletion mutants (red line) in LB Medium containing 512ug/ml SMX**.** Curves are generated by GraphPad Prism 10.3.0 and represent the mean ± standard deviation of three independent biological replicates, each with three technical replicates**.**

**Supplementary Figure S8:** Growth curves of mutants with *sul3* background in the presence of SMX**.** Growth kinetics of sulfa-resistant parent strain *MG1655/pACYC184_Sul3(SULFA^R^*) (green line) compared to its respective ten deletion mutants (red line) in LB Medium containing 1024ug/ml SMX**.** Curves are generated by GraphPad Prism 10.3.0 and represent the mean ± standard deviation of three independent biological replicates, each with three technical replicates.

**Supplementary Figure S9:** Growth curves of mutants with *dfrA1* background in antibiotic-free conditions**.** Growth kinetics of TMP-resistant parent strain *MG1655/pACYC184_dfrA1(TMP^R^*) (green line) compared to its respective ten deletion mutants (red line) in LB Medium. Curves are generated by GraphPad Prism 10.3.0 and represent the mean ± standard deviation of three independent biological replicates, each with three technical replicates.

**Supplementary Figure S10:** Growth curves of mutants with *dfrA5* background in antibiotic-free conditions**.** Growth kinetics of TMP-resistant parent strain *MG1655/pACYC184_dfrA5(TMP^R^*) (green line) compared to its respective ten deletion mutants (red line) in LB Medium. Curves are generated by GraphPad Prism 10.3.0 and represent the mean ± standard deviation of three independent biological replicates, each with three technical replicates.

**Supplementary Figure S11:** Growth curves of mutants with *dfrA12* background in antibiotic-free conditions. Growth kinetics of TMP-resistant parent strain *MG1655/pACYC184_dfrA12(TMP^R^*) (green line) compared to its respective ten deletion mutants (red line) in LB Medium. Curves are generated by GraphPad Prism 10.3.0 and represent the mean ± standard deviation of three independent biological replicates, each with three technical replicates.

**Supplementary Figure S12:** Growth curves of mutants with *dfrA5* background in the presence of TMP. Growth kinetics of TMP-resistant parent strain *MG1655/pACYC184_dfrA5(TMP^R^*) (green line) compared to its respective ten deletion mutants (red line) in LB Medium containing 1500ug/ml TMP**.** Curves are generated by GraphPad Prism 10.3.0 and represent the mean ± standard deviation of three independent biological replicates, each with three technical replicates**.**

**Supplementary Figure S13:** Growth curves of mutants with *dfrA12* background in the presence of TMP**.** Growth kinetics of TMP-resistant parent strain *MG1655/pACYC184_dfrA12(TMP^R^*) (green line) compared to its respective ten deletion mutants (red line) in LB Medium containing 1500ug/ml TMP**.** Curves are generated by GraphPad Prism 10.3.0 and represent the mean ± standard deviation of three independent biological replicates, each with three technical replicates.
